# Limited Joint Mobility Progression in Type 1 Diabetes: A 15-Year Follow-Up Study

**DOI:** 10.1155/2018/1897058

**Published:** 2018-05-02

**Authors:** Javier Labad, Antoni Rozadilla, Paula Garcia-Sancho, Joan M. Nolla, Eduard Montanya

**Affiliations:** ^1^Endocrine Unit, Hospital Universitari Bellvitge, L'Hospitalet de Llobregat, Barcelona, Spain; ^2^Parc Tauli Hospital Universitari, I3PT, Universitat Autònoma, CIBERSAM, Barcelona, Spain; ^3^Rheumatology Section, Hospital Universitari Bellvitge, L'Hospitalet de Llobregat, Barcelona, Spain; ^4^Institut d'Investigació Biomedical de Bellvitge (IDIBELL), Barcelona, Spain; ^5^Department of Clinical Sciences, University of Barcelona, L'Hospitalet de Llobregat, Barcelona, Spain; ^6^CIBER de Diabetes y Enfermedades Metabólicas Asociadas (CIBERDEM), Barcelona, Spain

## Abstract

**Objective:**

To assess the evolution of joint mobility over a period of 15 years in type 1 diabetic patients and healthy controls and to determine whether microalbuminuria is associated with a different evolution of joint mobility.

**Methods:**

Joint mobility of hand and wrist was determined in 63 patients with type 1 diabetes and 63 healthy subjects. Fifteen years later, 37 (58.7%) diabetic patients and 16 (25.4%) healthy subjects were studied again. Joint mobility was assessed with the Prayer sign and by measuring the angle of maximal flexion of the fifth and third metacarpophalangeal (MCP) joints and wrist. Patients with diabetes were visited 2–4 times every year with regular assessment of glycated hemoglobin (HbA1_c_), urinary albumin excretion (UAE), and ophthalmoscopy.

**Results:**

Fifteen years after the initial exam, diabetic patients showed reduced flexion of the fifth MCP joint (82.6 ± 5.8 versus 76.0 ± 6.4 degrees, *p* < 0.001) and wrist (75.9 ± 8.1 versus 73.2 ± 7.4 degrees, *p* = 0.015) compared to baseline examination. Joint mobility did not change significantly in healthy subjects. Patients with microalbuminuria showed greater reduction in hand joint mobility than diabetic patients with normal UAE or than healthy subjects (*p* < 0.001).

**Conclusions:**

In type 1 diabetic patients, the severity of LJM progresses with time, and the progression is enhanced in patients with microalbuminuria.

## 1. Introduction

Limited joint mobility (LJM), a nonpainful contracture of finger joints, is the most common hand abnormality in diabetes, but it has received little attention in clinical research and care [1–3]. LJM usually begins at the fifth interphalangeal joints and extends radially, affecting other interphalangeal and metacarpophalangeal joints. Long-term glycemic control influences the onset of LJM, and the incidence of LJM is greater in patients with a poor metabolic control [4, 5]. Although some studies have shown that improvement in standards of glycemic control and diabetes care has reduced the prevalence of LJM [6, 7], the Diabetes Control and Complications Trial (DCCT) could not show a consistent association between intensive insulin therapy and the elements of cheiroarthropathy [5]. The prevalence of LJM has been reported to increase with diabetes duration [5, 8]. However, age may be a confounding factor in the increased prevalence of LJM with longer diabetes duration since joint mobility deteriorates with aging [9, 10].

It has been shown that in elderly patients, diabetes adds a negative effect on LJM [9]. However, an association between LJM with diabetes duration as age increases has not been established [9]. Moreover, the few prospective studies that have determined joint mobility in diabetic subjects have not included a control group of healthy subjects [11–13]. Thus, it is not well established whether the evolution of LJM with aging is different in patients with diabetes and in the general population.

Cross-sectional studies have reported an association between LJM and microvascular complications [5, 7, 14–19], and the presence of LJM is considered a useful clinical tool to identify patients at increased risk for developing diabetic microvascular complications. However, prospective studies have yielded controversial results. In adults, the role of LJM in the prediction of microvascular diabetic complications has not been confirmed [11, 12], whereas in children, LJM is associated with an increased risk of microalbuminuria [13]. In a previous study [14], we found that hand joint mobility was limited in type 1 diabetic patients compared with control subjects and that LJM was associated with microalbuminuria. In the current follow-up study, performed 15 years later after the initial evaluation, we aimed to determine whether the evolution of joint mobility is different in diabetic and nondiabetic subjects and whether it is modified in the presence of albuminuria.

## 2. Methods

### 2.1. Subjects

In a previous study, we measured joint mobility in 63 patients with type 1 diabetes recruited from the outpatient clinic and in 63 age- and sex-matched healthy subjects [14]. The inclusion/exclusion criteria were described in the baseline study [14]. For the current study, the cohorts of diabetic patients and healthy subjects were contacted 15 years later and invited to participate in this follow-up study. Thirty-seven type 1 diabetic patients (58.7% of the initial cohort) and 16 healthy subjects (25.4% of the initial cohort) were accepted to participate and were included in the study. The clinical characteristics of patients and control subjects included in the follow-up were not significantly different from those of the initial study.

### 2.2. Joint Mobility

Joint mobility was assessed by the same rheumatologist (AR) that performed the initial assessment and using the same methodology. He was unaware of the metabolic control of patients, presence of diabetic complications, and individual values of the baseline joint mobility assessment. Joint mobility was determined qualitatively with the Prayer sign and quantitatively by measuring the maximal flexion of the fifth and third metacarpophalangeal (5MCP, 3MCP) joints and wrist, as previously described [14]. The maximal extension of the 5MCP and 3MCP joints was not recorded since the values, in both diabetic and control subjects, were inconsistent with those of baseline examination. In brief, the Prayer sign was defined as positive when subjects were unable to oppose palmar surfaces at any interphalangeal or MCP joint. To measure the angle of active maximal flexion of the fifth MCP joint, the fourth finger was fixed on a flat surface and the subject was asked to actively perform the flexion of the fifth finger at the level of the MCP joint. The angle of maximal flexion was measured with a goniometer and expressed in degrees using the zero method. To measure the mobility of the third MCP joint, the second finger was fixed. The arm and forearm were fixed in complete extension to measure the angles of active maximal flexion and extension of the wrist. Both hands were evaluated in all subjects, and the mean value between left and right measurements was used for statistical calculations.

### 2.3. Metabolic Control and Microvascular Complications

Medical records of the 15-year follow-up period were reviewed. Five years after the baseline study, the method to evaluate glycated hemoglobin was changed from HbA_1_ to the more specific HbA_1c_. Therefore, the metabolic control of the follow-up period was calculated as the mean HbA_1c_ value of the final 10 years. Diabetic retinopathy was assessed by direct ophthalmoscopy through dilated pupils. Urinary albumin excretion (UAE) was determined in 24 hour sterile urine. Albuminuria was defined as UAE greater than 30 mg/24 h in two consecutive samples or in two of three consecutive samples.

### 2.4. Statistical Analysis

Data were analysed using SPSS 15.0. Nonparametric tests Wilcoxon and Kruskal-Wallis were used to compare continuous data between groups. Kruskal-Wallis test was used to analyse differences in flexion among control group, diabetic patients with normoalbuminuria, and diabetic patients with microalbuminuria. Post hoc analysis between two groups was performed with the Wilcoxon test. Fisher exact test was used to compare categorical data between groups. The relationship between continuous variables was assessed with Pearson's correlation coefficient. A *p* value <0.05 (two-tailed) was considered to be significant.

## 3. Results

Clinical characteristics of diabetic patients at baseline and at follow-up are shown in Table 1. Nondiabetic control subjects had a similar sex distribution (43.8% male patients) than diabetic patients but were slightly older (33.8 ± 10.9 versus 27.4 ± 12.5 years at baseline). Diabetic patients showed LJM both at baseline and at follow-up compared with control subjects (Table 2). Joint mobility deteriorated with time in diabetic patients that showed reduced flexion of 5MCP joint and wrist at the end of follow-up compared to baseline (Table 2). In contrast, no significant differences were detected between baseline and follow-up in control healthy subjects. The prevalence of Prayer sign was the same at baseline and at follow-up examination and in the range of what has been described in diabetic and in nondiabetic subjects [20, 21].

**Figure 1:**
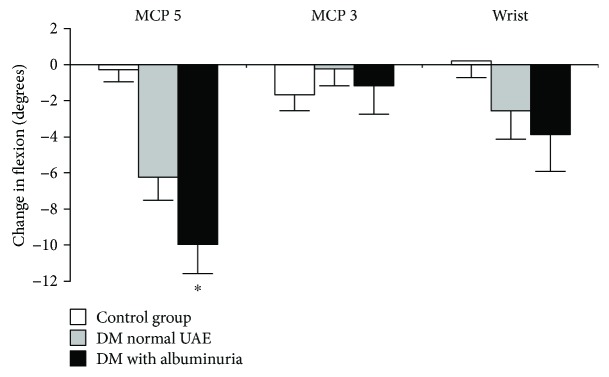
Change in hand joint flexion in diabetic patients (DM) with (*n* = 16) and without (*n* = 21) albuminuria and in healthy subjects (*n* = 16) over 15 years. DM: diabetes mellitus; MCP: metacarpophalangeal. Error bars represent ±1 SE. ^∗^*p* < 0.001 between groups (Kruskal-Wallis comparison). Post hoc between-group comparisons: control group versus diabetic patients without microalbuminuria, *p* = 0.001; control group versus diabetic patients with microalbuminuria, *p* < 0.001; diabetic patients without microalbuminuria versus diabetic patients with microalbuminuria *p* = 0.059.

Patients with albuminuria showed greater reductions in joint mobility after 15 years of follow-up than diabetic patients with normal UAE or than healthy subjects (Figure 1). In contrast, the presence of retinopathy was not associated with greater reductions in joint mobility. Changes in joint mobility were not associated with age, smoking, hypertension, dyslipidemia, and duration of diabetes or mean HbA_1c_ levels. There were gender differences in the reduction in wrist joint mobility, with a greater reduction in male patients (males: −7.1 ± 7.2 versus females 0.8 ± 7.0, *p* = 0.005). There were no significant differences in HbA_1c_ levels between patients with or without a Prayer positive sign.

## 4. Discussion

In this study, we show that, over a period of 15 years, joint mobility deteriorated significantly in patients with type 1 diabetes but not in healthy control subjects. Moreover, diabetic patients with albuminuria showed a more severe deterioration of joint mobility than those with normal UAE or than control nondiabetic subjects. The changes of joint mobility did not correlate with age, duration of diabetes, or metabolic control and were not modified by the presence of diabetic retinopathy.

To the best of our knowledge, this is the first study prospectively comparing the evolution of joint mobility in diabetic patients and nondiabetic subjects. The high prevalence of LJM in type 1 diabetes is well established [5], but longitudinal studies of joint mobility are scarce, and it is not known whether the evolution is different in diabetic patients and the general population. The presence of a control group of nondiabetic subjects with similar age is essential for this analysis, since age is associated with LJM [9]. In our initial study [14], we found that joint mobility was reduced in patients with type 1 diabetes compared with healthy controls. We now show that 15 years later, joint mobility deteriorated significantly in diabetic patients but did not change significantly in control subjects, indicating that the progression of limited joint mobility was specifically associated with the presence of diabetes and was not due to aging.

Type 1 diabetic patients with albuminuria showed a higher reduction in joint mobility after 15 years of follow-up compared with diabetic subjects with normal UAE and with control healthy subjects. In our initial cross-sectional study, we found that LJM was associated with microalbuminuria [14], an observation that has been subsequently confirmed by Amin et al. in a large longitudinal study [13]. We have now found that the presence of albuminuria is associated with a more severe progression of LJM. A plausible biological link between microalbuminuria and LMJ has been proposed based on a common role of advanced glycation end products in the development of LJM and diabetic complications including albuminuria [20–22]. However, other prospective studies have failed to show a relationship between microalbuminuria and LJM [11, 12]. Differences in the characteristics of diabetic patients and in the duration of follow-up may account for the discrepancy in the results. The shorter follow-up period of previous studies may have been insufficient to detect the association between microalbuminuria and poor evolution of LJM.

Our study has some limitations. First, the sample size was reduced from our initial study. Dropout rates may be accounted by the difficulty in contacting the subjects after this long follow-up period of 15 years and in particular the control subjects. Nevertheless, diabetic and control groups remained well matched in age and sex, and more than half of the initial diabetic population participated in the follow-up evaluation. Second, the extension mobility of the joints could not be evaluated. Extension of the 5MCP and 3MCP was already significantly reduced in diabetic subjects at baseline, and we may speculate that the follow-up extension measurements would have shown similar evolution than the joint flexion. Third, we focused on the diabetic microvascular complications that had been analysed in the baseline study, retinopathy, and in particular albuminuria, and we did not assess diabetic neuropathy. Reduced joint mobility in the hand has been associated with a decline in mobility in the ankle and is considered a risk factor for food ulceration in diabetic patients [21, 23]. However, diabetic neuropathy has not been consistently related to LJM [24]. Finally, the evaluation was not fully blinded regarding the presence of diabetes.

In summary, the present study shows the progressive character of LJM in type 1 diabetic patients and underscores the relationship between albuminuria and LJM. Periodic evaluation of joint mobility should be considered in patients with type 1 diabetes, particularly when albuminuria is present.

## Figures and Tables

**Table 1:** Clinical characteristics of diabetic patients (*n* = 37).

	Baseline	Follow-up
Age (years)	27.4 (12.5)	42.9 (12.3)
Female sex	19 (51.4)	19 (51.4)
Age at diabetes diagnosis (years)	19.8 (10.6)	19.8 (10.6)
Duration of diabetes (years)	7.7 (6.1)	22.6 (5.9)
BMI (kg/m2)	23.0 (3.0)	26.6 (4.0)
Systolic blood pressure (mmHg)	118.8 (14.6)	126.6 (19.5)
Diastolic blood pressure (mmHg)	71.1 (9.1)	72.1 (10.9)
HbA_1_ (%)	11.2 (1.7)	
HbA_1c_ (%)		8.1 (1.5)^a^
Insulin dose (U/kg)	0.75 (0.18)	0.83 (0.17)
Smoking	5 (13.5)	8 (21.6)
Hypertension	6 (16.2)	9 (24.3)
Dyslipidemia	2 (5.4)	8 (21.6)
Retinopathy	13 (35.1)	18 (48.6)
Microalbuminuria	7 (18.9)	16 (43.2)^b^

Values are mean (SD) or number of patients (%). BMI: body mass index; ^a^mean HbA1c values of the last 10 years of follow-up; ^b^two patients showed macroalbuminuria at the end of follow-up.

**Table 2:** Joint mobility in diabetic patients and healthy control subjects.

Joint mobility (degrees)	Patients with diabetes (*n* = 37)		Control group (*n* = 16)	
	Baseline	Follow-up	Baseline	Follow-up
Fifth metacarpophalangeal flexion	82.6 (5.8)^††^	76.0 (6.4)^∗∗^	86.0 (6.5)	85.8 (6.7)
Third metacarpophalangeal flexion	86.1 (3.8)^∗^	85.2 (4.4)	89.8 (5.8)	88.0 (5.8)
Wrist flexion	75.9 (8.1)^†^	73.2 (7.4)^∗^	76.3 (8.5)	76.5 (9.2)
Positive Prayer sign, *n* (%)	18 (48.6)^∗^	18 (48.6)^∗^	2 (12.5)	2 (12.5)

Values are mean (SD), except for Prayer sign (number and percentage) ^∗^*p* < 0.05, ^∗∗^*p* < 0.01 between patients and controls at each time point (Wilcoxon test for independent samples or Chi-square test [Prayer sign comparison]). ^†^*p* < 0.05, ^††^*p* < 0.001 between baseline and follow-up in patients or controls (Wilcoxon test for paired samples).
